# Mind–body therapy for cardiometabolic risk in U.S. middle-aged Black adults: a scoping review

**DOI:** 10.3389/fpubh.2025.1480369

**Published:** 2025-02-20

**Authors:** Danielle A. Martin, Jane Hook, Sunny Wonsun Kim, Linda Larkey, Rebecca E. Lee

**Affiliations:** ^1^Edson College of Nursing and Health Innovation, Arizona State University, Phoenix, AZ, United States; ^2^Center for Health Promotion and Disease Prevention, Arizona State University, Phoenix, AZ, United States

**Keywords:** scoping review, mind–body therapy, cardiometabolic risk factors, middle-aged black adults, psychosocial stress

## Abstract

**Background:**

In the U.S., Black adults do not achieve the same life expectancy as their White counterparts, and this is attributable in large part to the development of cardiovascular disease (CVD). Mind–body therapy (MBT) interventions demonstrate improvements in cardiometabolic risk (CMR) factors that promote CVD, with increased feasibility and acceptability in the general population. Less known is the feasibility, acceptability, and evidence of reduction in CMR factors in the U.S. Black population with MBT.

**Purpose:**

This study aimed to synthesize the current state of research regarding MBT on CMR factors in middle-aged U.S. Black adults and identify gaps in the literature. Research Question 1: What types of studies have been conducted (study design, theoretical framework, and cultural relevance)? and Research Question 2: What is the feasibility and acceptability and effectiveness of MBT in Black adults for CMR reduction?

**Methods:**

Following PRISMA-ScR guidelines, a review of three databases was conducted. Our inclusion criteria were articles that (1) describe empirical research; (2) assessed a MBT intervention in middle-aged (35–64) adults with a minimum of 60% Black adult participants for CMR reduction; and (3) written in English. Independent reviewers selected articles for inclusion and data extraction, with a third reviewer providing consensus.

**Results:**

Fourteen articles met the eligibility criteria (*n* = 14). Characteristics included randomized controlled trials (8, 57.1%); single-arm (3, 21.0%); mixed methods (3, 21.0%); sample size (17–375); mean age range 43–64; female (6, 42.8%); theoretical framework (4, 28.6%); culturally adapted (7, 50.0%); and studies demonstrating feasibility and/or acceptability (7, 50.0%). Of the seven articles assessing CMR physiologic factors, five studies observed significant improvement. For the 11 studies assessing CMR psychological factors, 6 studies had statistically significant results and 3 studies identified trends toward positive statistical outcomes.

**Implication:**

A growing body of literature across research stages demonstrating acceptability, and feasibility, and evidence of effectiveness for selected outcomes of MBT in middle-aged Black adults with CMR factors shows promise. Future research recommendations include greater recruitment of Black men for MBT studies, larger sample sizes, and utilizing culturally adapted interventions for engaging Black adults in MBT for reduced CMR factors.

## 1 Introduction

U.S. Black adults do not achieve the same life expectancy as their white counterparts by an average of 4.1 years, primarily due to the higher incidence of cardiovascular disease (CVD) (1). While the rate of CVD, coronary artery disease, stroke, and peripheral vascular disease is decreasing in the overall population, the rate of decrease is not commensurate among Black adults. As of 2019, Black adults have a significantly higher risk of mortality (30%) compared to their White counterparts (2). Despite identification of this disparity and subsequent development of risk factor treatment protocols, CVD deaths in the Black population remained 30% higher than non-Hispanic Whites as of 2019 at last calculation according to the National Vital Statistics Reports (1). Earlier age of CVD diagnosis (50 years ±15 years) in Black adults has been self-reported compared to their White counterparts (3). It is, therefore, imperative to identify effective interventions for CVD and CVD prevention in the Black middle-age population.

Cardiometabolic diseases comprise the primary contributors to CVD (4). To prevent CVD effectively, it is crucial to reduce cardiometabolic risk (CMR) factors. These factors include obesity (specifically central adiposity) (5), diabetes, and sedentary lifestyle which are associated with altered inflammatory profiles, dyslipidemia, and hypertension, known precursors to coronary artery disease and stroke (6). Psychological stress is another significant CMR (6, 7). Psychosocial stressors for Blacks, in addition to universal life stressors, also include environmental stressors such as stereotyping, exposure to racial violence, effects of day-to-day discrimination, and socioeconomic status (6, 8, 9). All CMR factors can be potentially amenable through intervention.

Research on mind–body therapies (MBTs) has demonstrated the feasibility and acceptability in the general population, showing improvements in the modifiable CMR factors: body weight, blood pressure, smoking reduction, and a decrease of psychosocial stress (7, 10). The American Heart Association has acknowledged one such mind–body therapy, meditation, as a “reasonable adjunct” to established CMR reduction protocols (11). However, the body of literature regarding MBT specifically for Black midlife adults is in various stages of progress, providing limited knowledge about feasibility, acceptability, and effectiveness of this intervention. Addressing the potential of cultural impact on uptake and effects of such programs is also of concern. The purpose of this review is to review the scope (types of studies, trends in outcomes achieved, and cultural frameworks) of published research addressing feasibility and effectiveness of mind–body therapies on CMR factors in middle-aged Black adults.

Mind–body therapies (MBTs) encompass interventions that generally focus on present moment awareness, incorporate breathing techniques, and may include movement (12). MBTs fall under the category of intervention known as “complementary and alternative medicine” (13). Examples of MBT include mindfulness, meditation, and meditative movement practices such as yoga, tai chi, or qigong (13). Systematic reviews have shown the potential of these practices in improving blood glucose levels, reducing waist circumference (an index of adiposity in body composition) and body weight, and lowering blood pressure as well as alleviating psychosocial stress and or improving resiliency (10, 12, 14). The National Center for Complementary and Integrative Health’s statistics has indicated a 3-fold increase in MBT of meditation practices by American adults over the 5-year period between 2012 and 2017 alone (10).

There are only two previous systematic reviews of mind–body therapies focused specifically on Black women, reporting on RCTs or pilot studies. One review focused only on hypertension (15), and the other focused on chronic disease, metabolic syndrome, and CMR factors (16). Given the limited attention and comprehensiveness of this research, we provide a scoping review to more broadly examine the types of studies, progress toward a more robust body of literature in this area, and the current body of evidence in these MBT interventions. The goal of this review is to synthesize the current body of literature for MBT feasibility/acceptability in Black participants and determine potential effectiveness for CMR factor reduction to identify gaps for future work. Given the diversity of study design, MBT interventions, and CMR outcomes related to the MBT intervention, authors conducted a scoping review as the most suitable approach for this study. Through this process, we describe the state of the current published research and examine the feasibility and acceptability of the MBT interventions (and whether those criteria are tied to cultural adaptations of interventions, if any), and the current evidence for these interventions in reducing CMR factors in Black adults. The primary purpose of this study is to identify gaps in the literature and propose future research directions for both researchers and practitioners. Research Question 1: What is the current state of research on MBT interventions for CMR reduction among Black Adults? Research Question 2: What is known about the feasibility/acceptability and effectiveness of this research (and if/how related to cultural fit)? and Research Question 3: What is known about the MBT in Black adults for CMR reduction?

## 2 Methods

### 2.1 Search methods

**Figure 1:**
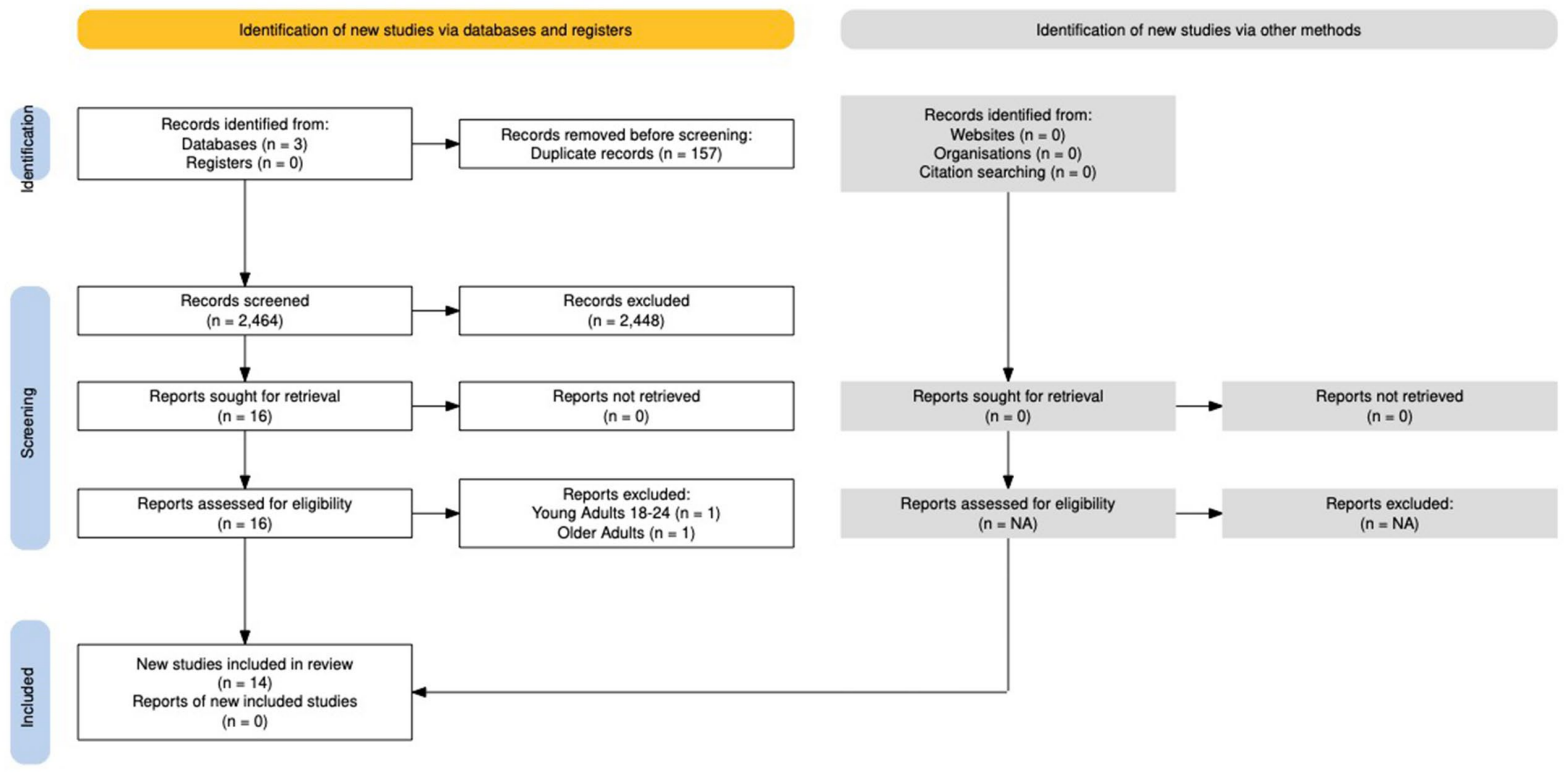
PRISMA-ScR flow map.

The Preferred Reporting Items for Systematic Reviews and Meta-Analyses for Scoping Reviews (PRISMA-ScR) guidelines were used to guide this review (17). The PRISMA-ScR flow diagram is included in Figure 1. Three English-language electronic databases (PubMed, CINAHL, and Scopus) were searched for articles published between 2012 and 2022, full text, and MeSH terms to identify mind–body therapy literature within the Black population. These databases were selected to capture the multidisciplinary nature of MBT studies.

The review time frame was chosen to identify changes in MBT use since 2012, extending the period examined by the American Heart Association for cardiovascular outcome disparities in Black adults. Prior to 2012, there was limited research on MBT within the Black community. This review looks to build on the prior literature, to also include Black men, and expand focus to include known psychosocial CMR factors.

Searches were conducted using the following search terms: African American, Black, Black American, cardiometabolic risk, cardiometabolic risk factors, heart disease, metabolic syndrome, meditative movement, breathing techniques, yoga, tai chi, qigong, meditation, breathing exercises, mindfulness-based stress reduction, mindfulness-based intervention, mind body therapies. Boolean terms AND OR were used in the search string. Reference lists of published systematic reviews were examined to identify additional sources not identified in the database searches. Studies targeting smoking were not sought because CVD, metabolic, and psychosocial CMR factors were the primary review interests.

#### 2.1.1 Inclusion criteria

The inclusion criteria for this review were determined before the literature search was initiated. Articles were included in this review if they were (a) implementing a mind–body therapy intervention (e.g., meditation, yoga, breathing exercises, tai chi, and qigong), (b) described as experimental, quasi-experimental, or mixed methods with an experiment aspect, (c) published between 1 January 2012 and 31 July 2022, (d) participants must be middle-aged (45–64) adults who (e) self-identify as Black or had 60% or more Black participants, and (f) published in English and conducted in the U.S.

#### 2.1.2 Exclusion criteria

Studies were excluded from this review if they were (a) systematic reviews, (b) observational studies, (c) qualitative only studies, (d) primary outcome was substance use, and (e) did not target any CMR factors or psychosocial stress (anxiety and depression) as outcomes.

### 2.2 Screening process

Articles found during our search process were initially screened by title and subsequently screened by abstract for eligibility by the first and second authors using Rayyan, “an intelligent research collaborative platform” (18).^1^ Articles that were potentially eligible were read in full and carefully examined by both authors independently to determine whether a given study met our predetermined eligibility criteria. Any discrepancies were reviewed together until consensus was reached. Any disagreements were resolved by additional authors for final decision.

### 2.3 Data extraction

A data extraction form was created to include study design, theoretical framework or conceptual model, cultural adaptation (if any), participant characteristics (i.e., age and gender), sample size, cardiometabolic outcome assessed, mode of mind–body therapy used, survey or instruments used, statistical analysis, and main results.

### 2.4 Reliability and quality assessment

The Grade Assessment, Development, and Evaluation (GRADE) criteria were used to assess each outcome across studies (19). For each study included in this review, two authors (DAM and JH) separately assessed for the included studies employing the GRADE assessment to determine evidence quality. The quality of evidence is ranked high, moderate, low, and very low based on initial assessment of study design (randomized control trial = high and non-randomized begin as a low rating due bias potential). Studies can be down- or upgraded based on the analysis of bias risk, inconsistency (heterogeneity), indirectness of evidence, impression in results, and high risk of bias in publication (19).

## 3 Search results

### 3.1 Selection of articles to include

This scoping review search results identified 2,621 records from CINAHL, PubMed, and Scopus. Duplicate studies (*n* = 157) were removed. DAM and JH conducted initial title and abstract screening of 2,464 studies. Common reasons for rejection included (1) population age not within eligibility range (2), outcomes assessed, and (3) not including mind–body therapy in intervention. There were 16 studies reviewed in full for final selection. Two articles were removed for the following reasons: (1) the study participants were young adults 18–24 and (2) the study participants were older adults 74 + years. After full text review, 14 articles met inclusion criteria and were included in the review (see PRISMA-ScR Figure 1). Data extracted from selected articles included in review are shown in Table 1.

**Table 1:** Extraction table MBT.

					Treatment type			
Author, year	Purpose	Model/framework	Sample size, mean age, gender, cardiometabolic risk outcome assessed (CMR)	Study duration/follow-up period	Control group	Experimental group	Instruments	Main results
Randomized control trials								
Bernstein et al., 2014^a^ (27)	Determined adherence with program and if the program helps reduce weight and blood glucose	Not indicated	Sample size, 27 (100% Black) Mean age, 56 Gender, F CMR, obesity, weight change, and diabetes	Intervention 6 weeks	Usual care	Lifestyle intervention MBT: meditation and guided imagery	Biometrics Waist circumference, weight, BMI, Cardiovascular BP, Framingham risk for 10-year risk of coronary disease, c-reactive protein Diabetes Fasting glucose, fasting plasma insulin, insulin resistance, A1c, HDL, LDL, total cholesterol, triglycerides Psychosocial stress PSS Physical activity (PA) Recent PA questionnaire Quality of life RAND SF-36 Dietary Habits ASA24, PI created food preparation questionnaire	92% class participation Trends of improvements in healthy cooking and eating habits were observed No statistically significant changes in weight, blood sugar, or other biometrics were observed
Burnett-Zeigler et al., 2019^b^ (29)	Examined distribution and variability of psychological outcomes in groups led by an experienced instructor compared to novice instructor	Not indicated	Sample size, 74 (71% Black) Mean age, 48.17 Gender, F CMR, depressive symptoms, depression, and stress	Intervention 8 weeks Follow-up 16 weeks	NA	Novice instructor vs. experienced instructor led modified MBSR “M-Body” MBT: meditation, toga, body scan	Psychosocial stress PSS IDS-SR DSSS Physical Activity WHODAS 2.0 Well being RPWB Mindfulness FFMQ	Experienced instructor group had a significant decrease in depressive symptoms from baseline to 16 weeks (*p* = 0.042, *d* = 0.84) Novice instructor group showed significant decrease in depressive symptoms from baseline to 8 weeks (*p* < 0.001, *d* = 1.05) and baseline to 16 weeks (*p* < 0.001, *d* = 0.83) Experienced instructor group had an increase in overall mindfulness from baseline to 8 weeks (*p* = 0.037, *d* = −0.88) and baseline to 16 weeks (*p* = 0.012, *d* = −1.06) Novice instructor group had significant increase in overall mindfulness from baseline to 8 weeks (*p* < 0.001, *d* = −0.67) and baseline to 16 weeks (*p* < 0.001, *d* = 0.68) Experienced instructor group had significant decrease in stress from baseline to 8 weeks (*p* = 0.014, *d* = 1.06) and baseline to 16 weeks (*p* = 0.015, *d* = 1.02) Novice instructor group had significant decrease in stress from baseline to 8 weeks (*p* < 0.001, *d* = 0.79) and baseline to 16 weeks (*p* < 0.001, *d* = 0.,95)
Cox et al. (26)	Evaluated a novel stress management group-based behavioral weight control program compared to traditional behavioral lifestyle program	Not indicated	Sample size, 44 (100% Black) Mean age, 44.5 Gender, F CMR, stress	Intervention 12 weeks	Lifestyle diabetes prevention program	Lifestyle intervention + stress MBT: guided relaxation, breathing techniques	Biometrics weight, height, BMI Psychosocial stress PSS, salivary cortisol	Retention rate of 86% Weight significantly decreased for both groups (*p* < 0.001) Weight loss significantly associated with the number of sessions attended (r = 0.31, *p* = 0.04), total diaries reported (*r* = 0.39, *p* = 0.01) From baseline to post-intervention women in both groups reported lower perceived stress (p = 0.01) no difference between groups A trend toward greater reduction in salivary cortisol was observed in the Lifestyle intervention + stress group (*p* = 0.20)
Mama et al., 2018^a,b^ (22)	Evaluated the feasibility and acceptability of Harmony and Health	Typology of adaptation	Sample size, 50 (100% Black) Mean age, 49.7 Gender, M&F CMR, depression, anxiety, stress, QoL, and physical activity	Intervention 8 weeks Follow-up 6 weeks	Wait list	Culturally adapted “Health and Harmony” MBT: yoga, meditation, breathing techniques	Biometrics BMI Psychosocial stress PSS, CES-D, Beck Anxiety Inventory, Positive & Negative Affect Scale Physical Activity IPAQ Wearable ActiGraph GT3X Quality of life RAND SF-36	Retention rate 80%; adherence rate 61.5%; satisfaction Rate 100% (assessed with 40% of participants) Trends suggest that participants in the intervention group reported greater improvements in self-reported physical activity with maintained improvements over time and psychosocial outcomes
Okhomina et al., 2018^a,b^ (32)	Compared retention and adherence rates between yoga, walking, and health education interventions	Not indicated	Sample size, 375 (100% Black) Mean age, 59.6 Gender, M&F CMR, cardiovascular disease	Intervention 24 weeks Follow-up 24 weeks	Health Education	3 yoga frequency groups (high, moderate, low) Guided walking group MBT: yoga, meditation, breathing techniques	Biometrics Waist circumference Cardiovascular BP Diabetes HDL, LDL, A1c	Retention 75.4%, pooled yoga group had the most participants complete the study (70.4%) Adherence (successful at 75%) was reached first by the pooled yoga program at week 12, followed by the education group at the 23. The moderate frequency yoga group achieved the highest adherence throughout the intervention, reaching 80.2% at week 24.
Schneider et al., 2012 (24)	Evaluated effects of transcendental meditation program in the secondary prevention of CVD	Not indicated	Sample size, 201 (100% Black) Mean age, 59 Gender, M&F CMR: cardiovascular mortality, BP, psychosocial stress factors, and lifestyle behaviors	Intervention transcendental meditation Follow-up 5.4 years	Health Education	Transcendental meditation MBT: meditation	Biometrics weight, height, BMI Cardiovascular BP Psychosocial stress CES-D, CMHI, Anger expression scale Physical Activity Modified leisure time physical activity questionnaire Dietary Habits Dietary food consumption questionnaire	A significant decrease in systolic BP was found in the transcendental meditation group compared to the control group 95% CI, −8.3 to −1.5 mm Hg; *p* = 0.01. A 5.4-year hazard risk was calculated and found transcendental meditation vs. control group had a significant reduction in myocardial infarction, stroke, and mortality risk (hazard ratio, 0.52; 95% confidence interval, 0.29–0.92; *p* = 0.025).
Schneider et al., 2019 (25)	Evaluated the effects of stress reduction with transcendental meditation on preventing LVH	Not indicated	Sample size, 85 (100% Black) Mean age, 52.8 Gender, M&F CMR, left ventricular mass index, systolic and diastolic blood pressure, anger, and perceived stress	Intervention 12- weeks Follow- up 6 months	Health Education	Transcendental meditation MBT: meditation	Cardiovascular BP, M-mode echocardiogram Psychosocial stress PSS, Anger expression scale, California self-evaluation scale 5-item subset, Personal scale	At follow-up LMVI and pulse rate decreased significantly for the transcendental meditation group compared to the control group (*p* = 0.040, *p* = 0.002 respectively). It was noted that systolic BP and diastolic BP decreased significantly within groups, not between. Perceived stress increased significantly in both groups, change not significant between groups
Woods–Gibscombè et al., 2019^a^ (28)	Examined the feasibility of conducting an randomized controlled trials of a novel mindfulness-based stress management program combined with diabetes risk-reduction education versus a conventional diabetes risk-reduction education program	Not indicated	Sample size, 68 (100% Black) Mean age, 52.66 Gender, M&F CMR: stress, QoL, diabetes, BMI, and physical activity	Intervention 8 weeks Follow-up 6 months	Conventional diabetes risk-reduction program	Adapted mindfulness-based stress reduction MBT: body scan, breathing techniques	Biometrics waist circumference, weight, height, BMI, hip circumference Diabetes HOMA-IR, A1c Psychosocial stress PSS Physical activity 7-day Physical Activity Recall Quality of life FACIT-Sp-Ex Dietary habits FHCRC_FFQ	Retention rate 90%, enrollment rate 79%, attendance rate 76.5% In the intervention group trends were observed in reduced BMI, calories, perceived stress, and dietary habits, and spiritual wellbeing
QUASI/single-arm								
Smith et al., 2015 (33)	Tested whether MBSR is effective in reducing stress and anxiety and in improving mindfulness and health-related quality of life	Not indicated	Sample size, 23 (87% Black) Mean age, 54.9 Gender, M&F CMR, depression and anxiety	Intervention 4 weeks	NA	Mindfulness-based stress reduction (MB SR) short form MBT: MBSR	Psychosocial stress PSS, GAD-7 Quality of life RAND SF-36 Mindfulness PMS	Self-reported anxiety reduced significantly (*p* = 0.005) Health-related quality of life subscales with statistical significance: Mental component summary (+9.1; *p* = 0.001), Social Functioning (+16.9; *p* = 0.003), Role Physical (+16.8; *p* = 0.016), and Mental Health (+15.6; P < 0.001), physical functioning (+6.6; *p* = 0.039), Vitality (+16.1; P = 0.001)
Waldron and Burnett-Zeigler, 2022^b^ (29)	Assessed the association between participation in a mindfulness-based intervention and post-traumatic stress symptoms	Not indicated	Sample size, 36 (88.99% Black) Mean age, 52.23 Gender, F CMR: post-traumatic stress, perceived stress, and depressive symptoms	Intervention 8 weeks	NA	Modified MBSR “M-Body” MBT: meditation, yoga, body scan	Psychosocial stress PSS, IDS-SR, DSM-5 (PCL-5), DSM-5 (LEC-5) Mindfulness FFMQ	From baseline to end of intervention: trauma symptoms significantly decreased t = 3.28, p = < 0.01, depressive symptoms decreased from a mean of 26.94 (SD = 12.32) to 20.11 (SD = 10.53), t = 3.48, *p* < 0.01, perceived stress reduced from a mean of 19.89 (SD =7.85) to 16.00 (SD = 7.86), mindfulness scores increased 10 points *p* < 0.01.
Chang et al., 2021^a^ (20)	Evaluated the feasibility and potential benefits of an 8-week qigong exercise on physical ability and function, balance, frailty, depression and anxiety, and spiritual wellbeing	Not indicated	Sample size, 17 (100% Black) Mean age, 64 Gender, M&F CMR, depression and anxiety	Intervention 8 weeks	NA	Qigong	Biometrics DXA scan Psychosocial stress HADS Physical Activity 6MWT, SPPB, Physical function domain of SF-36, PROMIS-CAT, FIFE Wellbeing RPWB Mindfulness Qigong intervention questionnaire	Retention rate, 88.2%, attendance rate ranged from 25 to 93.8% for participants. A trend toward improved anxiety (35% of participants) and depression scores (65% of participants) were observed
Mixed methods								
Hong et al., 2022^b^ (31)	Examined the association of mindfulness with depression stigma	Not indicated	Sample size, 31 (100% Black) Mean age, 51.9 Gender, F CMR, depressive symptoms and depression stigma	Intervention 8 weeks Follow-up 16 weeks	NA	Mindfulness- based stress reduction (MBSR) modified “M-Body” MBT: mindfulness, meditation, body scan, yoga	Psychosocial stress IDS-SR, DSSS, self reported past depression diagnosis Mindfulness FFMQ	A significant increase in mindfulness was observed from baseline to 8 weeks (p = 0.04) and baseline to 16 weeks (p = 0.01) Depressive symptoms decreased significantly from baseline to 16 weeks (p = 0.04) only
Zhou et al., 2017^a,b^ (23)	Tested the feasibility of a multiple-component lifestyle intervention & cardiometabolic risk-reduction program on diet, activity, & stress, using community-engagement principles	Social cognitive theory and theory of planned behavior	Sample size, 34 (100% Black) Mean age, 56.1 Gender, M&F CMR: obesity/weight change, stress, diet, and physical activity	Intervention 12 weeks	NA	Behavioral lifestyle program MBT: yoga, breathing techniques	Biometrics weight, height, BMI, % body fat Cardiovascular BP Diabetes A1c, LDL, HDL, total cholesterol Psychosocial stress PSS Wearable Body media fit armband	Post-intervention male participants reduced body fat % (33.8 +/− 2.6 to 28 +/− 2.6, *p* = 0.043), 41% of participants lost at least 5lbs, 14.7% had a body weight reduction of 5% or more HbA1c was significantly reduced (p = <0.001) as well as cholesterol levels (total *p* = 0.034; HDL p < 0.001; LDL not significant) Participants perceived stress was positively associated with adiposity levels (weight *p* = 0.006; BMI *p* = 0.013)
Johnson et al., 2014^a,b^ (21)	Tested the feasibility and acceptability of a culturally tailored, internet-based intervention	Social constructivist, Pender’s health promotion model	Sample size, 24 (100% Black) Mean age, 43.4 Gender, F CMR: metabolic syndrome	Intervention 4 weeks	NA	Yoga dance posture “yogic” MBT: yoga, breathing techniques	Biometrics waist circumference, weight, height, BMI Cardiovascular BP Physical activity IPAQ	Retention 71% of participants Acceptability of intervention observed in 100% of participants

^aFeasibility, acceptability, retention, and adherence assessed. bCulturally tailored intervention. HDL, high-density lipoprotein; LDL, low-density lipoprotein; BP, blood pressure; PSS, Perceived Stress Scale; RAND SF-36, RAND short-form health survey;ASA24, Automated self-administered 24-h recall; IDS-SR, Inventory of depressive symptoms- self-reported; DSSS, Depression self-stigma scale; WHODAS 2.0, WHO disability adjustment scale 2.0; RPWB, Ryff’s scale of psychological wellbeing; FFMQ, Five facet mindfulness questionnaire; CES-D, Center for epidemiological studies depression scale; IPAQ, International physical activity questionnaire; CMHI, Cook-Medley hostility inventory; GAD-7, Generalized anxiety disorder scale; PMS, Philadelphia mindfulness scale; PCL-5, PTSD checklist for DSM-5; LEC-5, Life events checklist for DSM-5; DXA scan, dual x-ray absorptiometry scan; HADS, Hospital anxiety & depression scale; 6MWT, 6-min walk test; SPPB, Short physical performance battery; PROMIS-CAT, NIH patient-reported outcomes measurement information system; FIFE, Frailty index for elders; HOMA-IR, Homeostasis model assessment of insulin resistance; FACIT-Sp-Ex, Functional assessment of chronic illness therapy, spiritual wellbeing expanded version; FHCRC FFQ, Fred Hutchinson cancer research center food frequency questionnaire.^

### 3.2 Study characteristics

Study characteristics of the eligible studies are presented in Table 2. The included studies were published between 2012 and 2022. All studies took place in the United States. Five (35.7%) were conducted in a health center, four (28.6%) were conducted on a university campus, two (14.3%) were conducted at a local church, other study locations included a local public school, online delivery (*N* = 1, 7.14% each), and a final study left location unspecified. The studies utilized several different designs to assess the effects of MBT interventions. Three studies (21.4%) were mixed methods utilizing an experimental arm to the design, four studies were single-arm/quasi-experimental (28.6%), and seven studies (50%) were randomized controlled trials. Four studies (28.6%) indicated the use of a theoretical framework or model in their study design which included Laters Model (20), Social Constructivist theory and Pender’s Health Promotion Model (21), Typology of Adaptation (22), social cognitive theory and theory of planned behavior (23) (Table 2).

**Table 2:** Study characteristics (*n* = 14).

	*N*	%
Age		
Range	43.34–64	
Mean	53.2	
Race		
100% AA	11	78.0%
Above 60% AA	3	21.4%
Gender		
Female	6	40.0%
Male and female	8	57.1%
Design		
RCT	8	57.1%
Mixed methods	3	21.4%
Single-arm/quasi	3	28.6%
Cultural tailoring		
Yes	7	50.0%
Study location		
Health Center	5	35.7%
Church	2	28.6%
University	4	28.6%
Local Public School	1	7.1%
Online	1	7.1%
Not indicated	1	7.1%
Theoretical framework		
used	4	28.6%
Outcomes assessed		
Obesity/weight change	5	35.7%
Depression and depressive symptoms	6	42.8%
Feasibility, acceptability, retention, and Adherence	7	50.0%
Anxiety	4	28.6%
Stress	7	50.0%
Metabolic syndrome	1	7.1%
Quality of life	2	28.6%
Left ventricular mass index	1	7.1%
Blood pressure	2	14.3%
Diabetes A1c	2	14.3%
Diet	5	35.7%
Physical activity	8	57.1%
Mind–body therapies		
Mind–body stress reduction (MBSR)	5	35.7%
Meditation	10	71.4%
Yoga	7	50.0%
Body scan	6	42.8%
Qigong	1	7.1%
Breathing techniques	7	50.0%
Guided therapy	2	14.3%

### 3.3 Participant characteristics

Participant characteristics are shown in the extraction table (Table 2). All participants were middle-age ranging from 43 to 64 years and had existing CMR factors. Across the 14 studies, 11 (78.5%) consisted of 100% Black participants, with 3 studies (21.4%) having 60% or more Black participants included. The sample size of participants in the 14 studies ranged from 17 to 375. All results of all participants were included in this review. Six studies (40%) included only women. In two studies, the participants currently had coronary heart disease (24) or the risk factor hypertension (25). In five studies, participants were identified as overweight/obese, one focused only on BMI (23), with two including women participants (26, 27), two including prediabetes (27, 28), three including stress/trauma (26, 28, 29), and one including low physical activity (22). In two studies, participants were Black women with depressive symptoms (30, 31). In one study, participants were Black women at risk for metabolic syndrome (21). In one study, participants were recruited from the longitudinal Jackson Heart Study (JHS) (32). The final two studies described participants as middle-aged and older Blacks (20) and inner-city adults (33).

### 3.4 Intervention and control group characteristics

Intervention and control group characteristics are summarized in Table 2. The length of mind–body therapy intervention duration varied across the 14 studies, ranging from 4 weeks (21, 33) to 24 weeks (32). A variety of delivery formats were trialed in the studies as shown in Table 2. The implementation of these interventions also varied, with involvement of different professionals. Three studies were guided by mental health professionals, specifically behavioral specialists (27), master’s level counselors (26), and psychologists (23). One study was led by a trained qigong instructor (20), two by certified yoga instructors (22, 32), two by certified meditation instructors (24, 25), two by mindfulness instructors (28, 33), one by a health educator (29), one by the research principle investigator (31), and one by both the research principle investigator and health educator (30).

The types of MBT interventions spanned from breathing techniques to full mindfulness-based stress reduction programs (Table 2). Seven types of mind–body therapies were examined within the 14 studies. Of the seven mind–body therapies used in the interventions, meditation was the most frequently utilized, followed by yoga, breathing techniques, mind–body scanning, adapted MBSR, guided imagery, and qigong. Of the study control conditions, two studies provided a lifestyle behavior intervention (26, 28), three provided health education (24, 25, 32), one provided usual care (27), and the remaining eight studies provided either a waitlist intervention or no comparative intervention (20–23, 29–31, 33).

Seven studies incorporated models for theoretical frameworks or cultural tailoring to design the intervention. Of the studies guided by a framework, only one of them used the framework to directly adapt existing intervention protocols to address cultural concerns, Davidson et al.’s Typology of Adaptation (22). Others used theories/models such as Social Constructivist and Pender’s Health Promotion Model (21), social cognitive theory, and theory of planned behavior (23) to guide and assess their interventions without directly referencing adaptations.

In terms of intervention modification, three studies (28, 30, 33) adjusted modified mindfulness-based stress reduction (MBSR) programs by reducing daily session duration, incorporating culturally relevant imagery, removing orientation and day-long retreats, and utilizing poetry from known Black women poets. In addition, two studies utilized faith-based wording (scriptures), music, and imagery (22, 23), while one study employed Western African dance as a part of its intervention design (21). Strategies aimed at participant retention included financial incentives for transportation, flexible session attendance, and fellowship between staff and participants (32). None of the studies did a side-by-side comparison of culturally adapted interventions to generic non-tailored intervention.

### 3.5 Quality of included studies: GRADE assessment

Reviewers DAM and JH reviewed and assessed the risk of bias using predefined criteria based on the Grade Assessment, Development, and Evaluation (GRADE) tool. GRADE assessment describes five appropriate categories of questions for randomized and non-randomized studies addressing: risk of bias or limitations in the study design and/or implementation, unexplained heterogeneity or inconsistency of results, indirectness of evidence, imprecision of results, high probability of publication bias (19). Quality was assessed at the outcome level. Outcomes deemed critical were CMR factor physiologic outcomes, psychological CMR factors, and feasibility/acceptability of the interventions.

Of the 14 studies, two of the eight RCTs were rated high certainty of evidence. There were four RCTs rated as low certainty and the remaining, and two RCTs were rated as moderate level of certainty. Reasons for downgrading the RCTs were lack of explanation of randomization procedures, under-powering of the study, lack of significance findings, or not testing CMR critical clinical outcomes. Ratings of 5 of the 6 non-randomized controlled trials (quasi, single-arm, or mixed methods) studies all remained low certainty rankings due to lack of justification for upgrade. One mixed methods study was upgraded to moderate due to its large effects on the clinically important outcome of adherence. Details regarding quality judgments for the 14 studies are presented in Table 3. The quality assessment for the included studies ranged from high- to very low-quality of evidence.

**Table 3:** Quality of studies using GRADE assessment.

Studies	Rating
RCT	
Bernstein et al. (27)	Low
Burnett-Ziegler et al. (30)	Low
Cox et al. (26)	Moderate
Mama et al. (22)	Low
Okhomina et al. (32)	Low
Schneider et al. (24)	High
Schneider et al. (25)	High
Woods-Giscombe et al. (28)	Moderate
Quasi/single-arm	
Smith et al. (33)	Low
Waldron and Burnett-Zeigler (29)	Low
Chang et al. (20)	Low
Mixed methods	
Hong et al. (31)	Low
Johnson et al. (21)	Moderate
Zhou et al. (23)	Low

## 4 Study results

### 4.1 Feasibility, acceptability, retention, and adherence

Fifty percent of studies (*n* = 7) tested feasibility, acceptability, and/or adherence. Feasibility was assessed in four studies (21–23, 28), and acceptability, defined as participant satisfaction, was assessed in one study using an *a priori* benchmark of 50% of participants rating the study intervention positively (21). Johnson, Taylor (21) conducted a trial of dance-modified yoga in middle-aged Blacks with CMR factors or diagnosed with metabolic syndrome and had as their primary aims testing feasibility, recruitment success, and adherence of the yoga technique. Using an a priori benchmark of 50% of respondents recruited, enrolled, and completing the study, Johnson, Taylor (21) reported 71% of participants completed all study components confirming their primary aims. In the study exit interviews, 59% of participants affirmed that the yoga intervention was acceptable. In the study of a mindfulness-based diabetes prevention intervention, Woods-Giscombe, Gaylord (28) had 79% of eligible participants complete 76.5% of intervention sessions and retained 90% of participants. There was a predetermined feasibility and adherence goal of 50% completion in a study of movement and faith-based mindfulness by Mama et al. (22). This goal was exceeded as 80% of participants completed an 8-week intervention targeting physical activity for CMR modification. Of the four studies that measured feasibility, three met their benchmarks (21, 22, 28).

Zhou et al. (23), combined multicomponent counseling (nutrition, mindfulness, and exercise) for their test of CMR factor reduction. This mixed methods study did not report retention or adherence statistics but did conduct focus groups during the study to enhance study acceptability to participants in real time.

Three studies considered attendance as a proxy for adherence and retention outcomes (20, 27, 32). Adherence was considered a key target by Bernstein et al. (27) in their multidimensional approach to diabetes which included mindfulness, meditation, and relaxation components; 100% of randomized participants in the intervention arm completed the study. Chang et al. (20) study of qigong in middle and older aged Blacks determined that qigong was acceptable as an intervention to their study participants based on self-reported response on the Qigong Intervention questionnaire. Okhomina et al. (32), in a longitudinal study of yoga versus guided walking and a health education control, also confirmed adherence and retention using yoga for CMR modification. This longitudinal study demonstrated that more than 70% of those in the yoga study arm completed a 6-month intervention and a 6-month follow-up.

Therefore, retention or adherence was confirmed in all four of those studies measuring those constructs. The one study that had a predetermined definition of acceptability met those specified standards (21).

### 4.2 Effects of mind–body interventions

The outcomes associated with CMR assessed in these studies included diabetes/A1c (2, 14.3%), obesity/weight control (3, 21.4%), metabolic syndrome (1, 7.1%), left ventricular mass index (blood pressure effect) (1, 7.1%), blood pressure (2, 14.3%), diet (5, 35.7%), physical activity (8, 57.1%), anxiety (4, 28.6%), stress (7, 50%), depression (6, 42.8%), and quality of life (2, 14.3%).

#### 4.2.1 Mind–body intervention affecting physiologic CMR reduction

In the seven studies addressing cardiometabolic physiologic outcomes, varied effects of MBT were observed. Three studies assessed obesity and weight change (22, 25, 26), two examined the effects of MBT on diabetes and hemoglobin A1c (26, 27), and three assessed blood pressure (23, 24, 31).

Notably, body weight and composition significantly improved in two studies that implemented a church-based lifestyle behavior program incorporating breathing, stretching, and yoga practices (22), as well as a lifestyle program combined with stress management techniques, including guided relaxation and mindfulness with diaphragmatic breathing (25). Zhou et al. (34) reported reductions in weight and hemoglobin A1c after the 12-week intervention. However, low-density lipoprotein (LDL) was not significantly changed (22). Cox et al. (25) found significant trends toward greater weight loss in their intervention group, despite their pilot study not being designed to detect significant differences. An adapted MBSR program also demonstrated a significant reduction in BMI in both groups at 3-month and 6-month follow-up (27).

In terms of blood pressure, significant reductions were noted in two interventions utilizing transcendental meditation (23, 24). Schneider et al. (23) reported significant decreases in systolic blood pressure in the transcendental meditation group compared to a health education control group, with a notable 5.4-year hazard risk reduction in myocardial infarction, stroke, and mortality.

Schneider et al. (24) found significant within-group reductions in both systolic and diastolic blood pressure among participants practicing transcendental meditation, although no significant difference emerged between the meditation and control groups (24).

While these studies suggest that mindfulness practices and meditation may benefit weight management, stress reduction, and cardiovascular health, they varied in design and scope. Some were small pilot studies that were not necessarily designed to detect significant changes, suggesting the need for further comprehensive research to determine the impacts of mindfulness-based interventions on cardiometabolic health.

#### 4.2.2 Mind–body intervention affecting psychological function

In studies that addressed psychological outcomes (*n* = 11), seven assessed stress (22, 23, 25, 26, 28–30), five assessed effects of MBT on depression or depression symptoms (20, 22, 24, 30, 31), three assessed anxiety (20, 22, 33), five assessed mindfulness (20, 29–31, 33), and two studies observed quality of life (22, 28).

##### 4.2.2.1 Stress

Several studies examining stress measurement utilized multiple methodological approaches, incorporating both validated self-report measures and physiologic markers to assess stress outcomes. The Perceived Stress Scale was identified as a primary tool utilized across studies. Culturally adapted mindfulness-based stress reduction interventions have shown significant reductions in perceived stress, while lifestyle change interventions varied in effectiveness among different participant groups.

Seven studies utilized the Perceived Stress Scale to measure stress (22, 23, 25–30, 33), one study utilized Perceived Stress Scale and salivary cortisol (26), and another used Perceived Stress Scale and Post-Traumatic Stress Disorder Checklist for the Diagnostic and Statistical Manual of Mental Disorders (DSM-5) (29).

Stress affect outcomes were variable across the seven studies. Two studies that tested modified MBSR interventions demonstrated a significant decrease in their perceived stress score (29, 30). Lifestyle change interventions showed to have a positive effect on stress. A study evaluating the effects of a lifestyle plus stress reduction intervention on Black women demonstrated improvements in perceived stress across all participants, and there was no difference between groups (26). In a church-based lifestyle intervention, baseline perceived stress was associated with baseline adiposity levels, weight, and BMI; however, this association did not remain throughout the intervention (23). In the adapted MBSR, perceived stress was significantly reduced at 3-month follow-up for the intervention group (28). Schneider et al. (35) observed a significant increase in perceived stress. These researchers posed that an increase in measured psychological variables for stress might be because of individual variation in stress perceptions and emotional responses. Authors found that this corroborated other transcendental meditation studies that demonstrated improved physiologic improvements without concurrent improvements in psychological variables.

Trends toward effects on stress were, however, found in three studies. A lifestyle plus stress group showed a trend toward greater reductions in salivary cortisol than in the control group (26). A short-form MBSR reduced stress by approximately 20% (33). Culturally adapted lifestyle intervention observed trends toward reduction of stress (22). These findings illustrate the complexity of stress measurement and interventions, highlighting the critical need for population-specific approaches that account for diverse individual and contextual factors affecting stress experiences and responses.

##### 4.2.2.2 Depression, anxiety, mindfulness, and quality of life

Researchers utilized multiple validated measures and self-reported surveys to assess psychological and wellbeing indicators, including depression, anxiety, mindfulness, and quality of life through various standardized questionnaires and assessment tools. Several validated measures and self-reported surveys were used to assess depression, anxiety, quality of life, and mindfulness. Depression was assessed across studies utilizing five different tools: Center for Epidemiological Studies of Depression Scale, Inventory of Depressive Symptoms Self-Reported, Depression Self Stigma Scale, Hospital Anxiety and Depression Scale, and self-reported past depression diagnosis. Across the four studies assessing anxiety, Generalized Anxiety Disorder, Beck Anxiety Inventory, and Hospital Anxiety and Depression Scale were utilized. Mindfulness was assessed using either the Five Facet Mindfulness Questionnaire or the Philadelphia Mindfulness Scale. Quality of life was assessed using either the Health-Related Quality of Life 36-item short form or Functional Assessment of Chronic Illness Therapy, Spiritual Well-Being Expanded Version.

Three studies employing modifications of the MBSR reported significant decreases in depression scores and improvements in mindfulness indices at a level of statistical significance following the intervention (29–31). Short-form MBRS was positively associated with a reduction in anxiety (*p* = 0.005) and an increase in quality of life, along with participants improving their mindfulness scores by 2 points from baseline to follow-up showing a trend toward statistical significance (33).

A study utilizing qigong exercise found that participants showed improved depression and anxiety change scores from baseline to follow-up, illustrating a trend toward significance (20). Mama et al. (22) identified positive trends in depressive symptoms from baseline to 6-week post-intervention follow-up. Schneider et al. (36) observed a decrease in depression in the transcendental meditation group. Researchers posit the lack of reduction in depression was due to an already low depression score at baseline for participants.

Two studies observed quality of life (22, 28). Mama et al. (22) was unable to demonstrate significant increases in quality of life. Woods-Giscombe et al. (28) observed the intervention group experiencing significant increases in total spiritual wellbeing at 6 months, contrasting with the control group who experienced significant decreases in spiritual wellbeing at 6 months.

Overall, multiple studies, particularly those employing modified mindfulness-based stress reduction interventions, reported significant decreases in depression scores, improvements in mindfulness indices, and positive trends in anxiety and depressive symptoms. Some studies demonstrated notable improvements in psychological wellbeing and spiritual health; others found more modest or mixed results, with researchers attributing variations to factors such as baseline participant characteristics and intervention design.

#### 4.2.3 Mind–body intervention affecting lifestyle change

The research examined lifestyle changes related to cardiovascular metabolic risk (CMR) factors, focusing on physical activity and dietary outcomes across multiple studies. Physical activity was measured in 57.1% (*n* = 8) of the mind–body intervention studies, whereas dietary outcomes were a target outcome in just over a third of studies (See Table 2). One study included alcohol consumption in their dietary data collection.

Physical activity was measured across studies using direct activity measurement and/or self-report surveys. Two studies used accelerometry as objective indices of physical activity: ActiGraph ™ (22) and Body Media Fit Armband ™ (23). Mama et al. (22) calculated moderate-to-vigorous physical activity from the accelerometer data, whereas Zhou et al. used the BodyFit wearable to capture daily step counts. Chang et al. (20) used physical functioning as their target activity outcome. Defined as mobility capability, they used the National Institutes of Health Patient-Reported Outcomes Measurement Information System Computerized Adaptive Short Form 36 which contains a 10-item mobility limitation subscale. The Chang et al. (20) study was the sole study conducting pre-post intervention 6-min walk tests to determine increased aerobic exercise capacity to quantify impact of improved physical functioning.

Six studies relied upon validated self-report tools to measure levels of physical activity. The International Physical Activity Questionnaire was used by two studies (21, 22). Additional self-report measures were the Recent Physical Activity Questionnaire (27), a Modified Minnesota Leisure Time Physical Activity Questionnaire, and the 7-day Physical Activity Recall (28). Schneider et al. (25) developed a custom physical activity diary to record duration of activity in minutes.

None of the studies demonstrated statistically significant improvements in physical activity levels. A trend toward a positive physical activity outcome was evident, however, in two studies with multicomponent interventions: qigong with musical accompaniment and pre-post relaxation sessions (20) and another with the combination of yoga, guided relaxation, music, breath focus, and spirituality sessions (22).

Five of the studies targeted dietary lifestyle change outcomes. Two of these studies modified existing diabetes programs (26, 27) which had physical activity and nutrition guidance to also include some aspects of cognitive behavioral training with mindfulness, and mediation and stress management (breath work and guided imagery), respectively. Bernstein et al. (27) combined physical activity recommendations and mindfulness training and used an investigator-designed food preparation intervention.

Schneider et al. (24) matched a MBT intervention (transcendental meditation) arm against a lifestyle change health education control arm which included diet instruction as did Woods-Giscombe et al. (28) who compared a traditional prediabetes lifestyle program to a mindfulness prediabetes intervention. Finally, Zhou et al. (23) single-arm study tested a multicomponent intervention that paired MBSR with diabetes risk reduction in a church setting.

The tools used to collect data to confirm dietary lifestyle changes were as follows: The National Institutes of Health/National Cancer institute Automated Self-administered 24-h dietary recall (27), the Dietary Food Consumption Questionnaire (24), Fred Hutchinson Cancer Research Center Food Frequency Questionnaire (28), and an investigator-designed questionnaire on food preparation (23). Food preparation specifics were methods of food preparation, ingredients, meals consumed in the home, mealtimes, and time spent in a meal. The Woods-Giscombe et al. (28) intervention demonstrated non-statistically significant decreases in both carbohydrates and lipids and therefore calorie intake in both the control (customary diabetes prevention group) and intervention arms (mindfulness + diabetes prevention) at 3- and 6-month follow-ups.

In summary, one of the dietary lifestyle studies had statistically significant changes in body fat with a trend toward lower sodium intake (23). Three of the other four studies also demonstrated a trend toward a decrease in BMI, body weight, or weight loss through their dietary lifestyle intervention. The research suggests that mind–body interventions may have potential for influencing lifestyle behaviors, although more comprehensive studies are needed to establish clear, consistent outcomes.

## 5 Discussion

This study used a scoping review strategy to summarize the state of the research, including types of studies, feasibility/acceptability evaluations, effectiveness of mind–body therapy interventions for reducing cardiometabolic risk factors in middle-aged Black adults, and evaluation of the attention to theory and/or culture in designing interventions. Identifying what has been covered in the research, and what one might learn from these studies’ types, intervention choices, feasibility evaluations, and outcomes are discussed with recommendations for what still needs to be addressed.

### 5.1 Types

Types of research and interventions reviewed in the current study are not unlike what previous reviews have noted, whether these were evidence or scoping reviews. For example, the seven types of mind–body therapies and their representation in the current review parallel the approximate proportions of studies going on within the Black population (12, 15) as well other populations (34, 37, 38).

### 5.2 Feasibility/acceptability

The large number of studies reporting primarily adherence and feasibility showed similar rates of adherence, feasibility, and attrition results as the prior reviews. As described in Results, rates of adherence ranged from 50 to 100% across the studies with an average adherence retention rate of 72.7%, regardless of length of study which varied from 4 weeks to 6 months and irrespective of the MBT intervention implemented. Similar rates of adherence (≥75%) were noted by Gravesande et al. (36) and Watt et al. (35) for online MBT interventions, completed in different adult populations and outcomes. Of the four studies in our review that assess feasibility, three met their benchmark goal. These findings parallel feasibility studies previously conducted in other adult populations (39). While half of the included studies focused on measuring feasibility or adherence, none demonstrated high attrition illustrating both feasibility and acceptability. Combined, the studies demonstrated strong feasibility, adherence, and or retention in the MBT participants indicating the possibility of scalability of adoption by Black adults. This overall finding corroborates the acknowledgment of the American Heart Association that MBTs are feasible and acceptable adjuncts to CMR factor reduction strategies (11).

### 5.3 CMR reduction outcomes

As described in the Results section examining physiologic changes in CMR reduction outcomes, analysis of the 14 selected articles revealed that mind–body therapies were shown to have an overall significant positive effect on blood pressure, pulse rate, BMI or weight reduction, hemoglobin A1c, and left ventricular mass index. Most illuminating among the physiologic findings was the overall reduction in mortality and reduction of cardiovascular events at 5-year follow-up using transcendental meditation. It should be noted that all studies showed trends toward positive improvements in the assessed outcomes. In general, the data are promising for continuing to build research in this area of study for middle-age Black adults with physiologic CMR conditions.

As described in the Results section, MBTs were shown to have an overall significant positive effect on psychological experiences such as perceived stress, depression, mindfulness, quality of life, and anxiety. We noted that one outcome measure was nearly consistently used across studies. Nine studies included the Perceived Stress Scale; as such, stress reduction has emerged as a salient target for CVD risk reduction (40).

While physical activity may be thought of by some as the MBT activity itself (for those MBTs that include movement), some have measured this as a target outcome because of the known effects of this lifestyle behavior on reducing CVD risk. Physical activity was measured as an “outcome” in 57.1% studies, interestingly half of which were studies in which the MBT was a practice that included movement. Although physical activity lifestyle changes did not significantly increase in any of the studies targeting this outcome, there were trends toward increases (and none showed decreases). It is interesting to note that X (number) studies did not measure physical activity as an outcome despite using a MBT that included a movement-based intervention. It could be concluded that participants in such interventions (e.g., yoga and qigong) will have increased their activity level to some degree unless they were already active prior to the study enrollment. Very few studies with movement-based MBTs exclude participants who are physically active. Since yoga and qigong have been shown to have a METS of 1.5–3.0, or 1.5 to 3 times the amount of resting energy expenditure, and their use is likely to result in some in improvement in physical activity levels, unless they entered the intervention with already-high levels. If participating in these MM practices for 30 min, 5 times/week, this approaches the physical recommendations for adults (450 of the 500 METS min/week) (41).

Given the heterogeneity of interventions used across studies, it would be difficult to identify indicators of a MBT “gold standard” to reduce CMR in Black adults. Most studies used MBT paired as a part of a multicomponent intervention. Intervention dose and duration also varied across studies. Follow-up time frames ranged from 4 weeks to 5 years. Schneider et al. (24) was the only study with long-term follow-up (5 years). The lack of long-term follow-up across studies failed to capture sustained intervention effects. Twelve of the 14 studies employed a health professional: behavioral health specialist (3), certified MBT instructor (7), and health educators (2) creating a barrier to wide scale adaptability. While these professionals were instrumental in delivering the interventions, their involvement could present a potential barrier to the widespread adoption of mind–body therapies as it may not be easily scalable in settings with limited access to such specialists. Finally, it is important to note that the proportion of male participants in the studies ranged from 4.3 to 35.35%, indicating lower participation levels for males than females. Thus, many of the studies present results that may not fully represent Black men for feasibility and/or outcomes, leaving gaps in our knowledge of MBT appropriateness or effectiveness in Black men.

### 5.4 Use of theory or cultural adaptation in design of interventions

Studies varied in their approach to adapting interventions for the population tested. Common strategies included community-based participatory research, cultural adaptation, and culturally informed settings. A notable example of a comprehensive culturally relevant approach involved a church community, where a research team of predominantly Black researchers worked alongside senior church members, and the pastor, guided by Davidson et al.’s Theory of Adaptation. This framework utilized an adaptation toolkit to develop the intervention (22). Davidson et al. (42) created a Toolkit of Adaptation Approaches designed to address cultural needs of the minority populations. The toolkit consists of three different items: 46-item Typology of Adaption Approaches, Pathway to Adaptation, and a decision-making tool titled RESET (Relevance, Evidence base, Stages of intervention, Ethnicity, and Trends). Mama et al. (22) utilized the Pathway to Adaptation, which included six categories: collaborated working, team, endorsement, materials, message, and delivery to create their culturally adapted intervention.

Another successful example of a culturally adapted intervention was conducted at a Federally Qualified Health Center, which provided transportation vouchers and reduced session times to accommodate participants’ needs along with modifying both wording and images to reflect the community (30).

No study specifically looked at a comparative difference between traditional (generic) MBT interventions and culturally adapted MBT interventions. Of the seven studies identified as culturally adapted, four demonstrated significant results in either physiologic health outcomes (23) or psychosocial health outcomes (29–31), with others showing trends toward significance (22).

Identifiable differences between traditional and culturally adapted MBT interventions in this review included sample size, primary outcome focus, study design, and duration. Traditional MBT interventions in this review tended to have larger sample sizes to adequately power to detect significance, while culturally tailored MBT interventions primary assessed feasibility (e.g., acceptability, retention, and adherence). Majority of these culturally adapted MBT interventions were not powered to test significance in secondary health outcomes. Moreover, study design played a role in the strength of the studies reviewed. Traditional MBT interventions mostly employed RCTs and/or longitudinal designs, where effect is gained over a significant amount of time utilizing several other influences. In contrast, many culturally adapted MBT interventions utilized mixed methods, offering qualitative aspect into the access barriers faced by the Black middle-age population in participating in MBT interventions.

While half of the studies did not utilize any form of adaptation, the studies that did use these strategies presented strong cases for creating a more accessible mind–body therapy that relates to Black adults to entice engagement in new forms of exercise to reduce CMR factors.

Future studies can improve culturally adapted MBT interventions by cultivating a people-centered research approach. Embracing a comprehensive cultural adaptation framework to guide community-based participatory research strategy which addresses community involvement, research design, recruitment methods, data collection protocols, and intervention implementation is advisable. Comparative studies examining generic versus culturally adapted approaches could provide valuable insight into the specific needs of the Black community with regard to improving research feasibility and acceptability of CMR reduction techniques.

### 5.5 Prior research

Our review corroborated other reviews which showed that there were an inadequate number of studies of MBT in our desired population (12, 15, 43). Our review was also in agreement that many “co-interventions” were bundled with MBT making the distinct impact of MBT difficult to assess. In addition, this review has also concluded that there are limited high-quality studies, and the risk of bias is substantial across retrieved studies. We also concluded that meta-analysis of MBT use in Black adults is limited currently given the heterogeneity of study characteristics (population, setting, intervention, and outcome measure(s)) and be powered RCTs who report statistical data such as effect sizes and confidence intervals for combining and comparison.

Where this review refutes other published reviews is that unlike, for example, a commentary on mindfulness research among Black Americans (43), we found a breadth of tailoring employed in our retrieved studies. We also found that the efforts to enact community-based population research strategies, in studies that employed them, were also exactingly described paving the way for replication of the researcher’s methods. Both the Mama et al. (22) and the Burnett-Zeigler et al. (30) studies were very prescriptive in their outlining cultural adaptation which facilitates the study of MBT in different settings and with different samples of individuals.

### 5.6 Limitations

In regard to limitations, we did not include studies targeting smoking which is a significant contributor to CVD. The choice to exclude studies with lower percentages of Black participants may have eliminated potential sources of information, but then, the information derived would have been less specifically relevant to our population of interest. Only four out of 14 studies indicated the use of a theoretical framework or model, suggesting study design weaknesses due to a lack of theoretical underpinning in the field of mind–body therapy interventions in the available studies. Another source of weakness in studies included was discussed in the GRADE analysis, indicating very few high-quality studies thus far. As such the available studies to review had some weaknesses making it more difficult to draw conclusions from the evidence base. An additional limitation to this study was the lack of studies specifically targeting males creating a knowledge gap regarding the acceptability or effectiveness of mind–body therapy interventions for modifying CMR in male Black populations. Of the 14 studies, 7 were early phase studies intended to power large-scale randomized controlled trial studies of the identified intervention in Black Adults. Finally, the choice of databases, and time frame, may have limited identification of pertinent studies. Despite these limitations, this study contributes substantially by not only synthesizing the evidence of mind–body therapy interventions and their impact on middle-aged Black CMR factors but that it also highlights potential research gaps, which can guide future meaningful research in the Black population.

### 5.7 Barriers and future direction

Biggers et al. (43) identified three broad areas of barriers to MBT participation in Black adults. These were identified as practical, representational, and alienation issues. A lack of awareness or limited knowledge of research participation presents a hurdle to considering joining a research study. Practical issues limit participation in research through making participation difficult or impossible. Representational issues contribute to the third category of alienation by creating a “foreign” feel for MBT rather than reinforcing a natural sense of being present in the moment. For some, MBT could present as a conflict to religious or spiritual beliefs and create dissonance for participants. Adapted messaging for recruitment including explanation of clinical research and participation should guide recruitment efforts. Centering MBT interventions in a community setting with like instructors, while addressing barriers such as transportation, childcare, and flexibility or convenience in intervention times, could enhance study participation, retention, and adherence factors. Focusing on representative researchers, interventionists to reflect the population could reduce enduring trauma of unethical medical experimentation. Finally, centering the adaptation of the MBT interventions themselves to be relevant, recognizable, and achievable would make MBT more palatable to participation in this population.

The persistent health disparities facing Black adults demand a multifaceted, culturally nuanced approach to reducing cardiometabolic risk factors. By leveraging implementation science and targeted interventions that recognize the complex interplay of biological, social, and environmental determinants, public health policies can more effectively address these critical health challenges (44, 45). The Healthy People 2030 objectives provide a strategic framework for focusing on key areas such as blood pressure reduction, cardiovascular health improvement, diabetes prevention, and physical activity promotion (46–49). Critically, these interventions must be developed through a lens of cultural appropriateness, drawing on diverse scientific disciplines and acknowledging the unique lived experiences of Black communities (44). The NIH’s strategic emphasis on inclusive research that understands the intersectionality of individual characteristics represents a promising path forward in addressing these deeply rooted health inequities (50). Ultimately, success will require a comprehensive, adaptive approach that respects the complexity of health disparities while remaining committed to meaningful, population-specific solutions.

## 6 Conclusion

The results from the scoping review suggest that there is a growing yet widely targeted body of literature addressing this type of intervention. This scoping review confirms that mind–body therapy interventions are deemed feasible and acceptable in this population. The current state of research further demonstrates that mind–body therapies show promise for decreasing CMRs risk factors in middle-aged Black adults. Future research should focus on conducting larger randomized control trials of MBT interventions in the Black community, development, and testing of culturally adapted MBT interventions to engage Black adults in mind–body therapies and conducting studies of male-specific mind–body therapy interventions.

## Data Availability

The original contributions presented in the study are included in the article/supplementary material, further inquiries can be directed to the corresponding author.

## Footnotes

^1^ https://www.rayyan.ai/

## References

[1] Xu JQ Murphy SL Kochanek KD Arias E. Deaths: Final data for 2019. Hyattsville, MD: National. Center for Health Statistics (2021). 32501199

[2] Office of Minority Health. *Heart disease and African Americans: U.S. Department of Health and Human Services*. (n.d.). Available at: https://minorityhealth.hhs.gov/omh/browse.aspx?lvl=4&lvlid=19.

[3] Lee K Huang X Wang MC Shah NS Khan SS. Age at diagnosis of CVDs by race and ethnicity in the U.S., 2011 to 2020. JACC Adv (Online). (2022) 1:100053. doi: 10.1016/j.jacadv.2022.100053, PMID: 36051947 PMC9432389

[4] CDC. *Heart disease and stroke* (2022). Available at: https://www.cdc.gov/chronicdisease/resources/publications/factsheets/heart-disease-stroke.htm.

[5] Klein S Allison DB Heymsfield SB Kelley DE Leibel RL Nonas C . Waist circumference and cardiometabolic risk: a consensus statement from shaping America's health: Association for Weight Management and Obesity Prevention; NAASO, the Obesity Society; the American Society for Nutrition; and the American Diabetes Association. Am J Clin Nutr. (2007) 85:1197–202. doi: 10.1093/ajcn/85.5.1197 17490953

[6] Powell-Wiley TM Poirier P Burke LE Després J-P Gordon-Larsen P Lavie CJ . Obesity and cardiovascular disease: a scientific statement from the American Heart Association. Circulation. (2021) 143:e984–e1010. doi: 10.1161/CIR.0000000000000973, PMID: 33882682 PMC8493650

[7] Levine GN Cohen BE Commodore-Mensah Y Fleury J Huffman JC Khalid U . Psychological health, Well-being, and the mind-heart-body connection: a scientific statement from the American Heart Association. Circulation. (2021) 143:e763–83. doi: 10.1161/CIR.0000000000000947, PMID: 33486973

[8] Carnethon MR Pu J Howard G Albert MA Anderson CAM Bertoni AG . CV health in African Americans: AHA. Circulation. (2017) 136:e393–423. doi: 10.1161/CIR.0000000000000534, PMID: 29061565

[9] Lewis TT Cogburn CD Williams DR. Self-reported experiences of discrimination and health: scientific advances, ongoing controversies, and emerging issues. Annu Rev Clin Psychol. (2015) 11:407–40. doi: 10.1146/annurev-clinpsy-032814-112728, PMID: 25581238 PMC5555118

[10] Kim DY Hong SH Jang SH Park SH Noh JH Seok JM . Systematic review for the medical applications of meditation in randomized controlled trials. Int J Environ Res Public Health. (2022) 19:1244. doi: 10.3390/ijerph19031244, PMID: 35162267 PMC8834867

[11] Levine GN Lange RA Bairey-Merz CN Davidson RJ Jamerson K Mehta PK . Meditation and cardiovascular risk reduction a scientific statement from the American Heart Association. J Am Heart Assoc. (2017) 6:2218. doi: 10.1161/JAHA.117.002218, PMID: 28963100 PMC5721815

[12] Johnson CC Sheffield KM Brown RE. Mind-body therapies for African-American women at risk for Cardiometabolic disease: a systematic review. Evid Based Complement Alternat Med. (2018) 2018:5123217. doi: 10.1155/2018/5123217, PMID: 29681975 PMC5846388

[13] National Center for Complementary and Integrative Health. *Complementary, alternative, or integrative health: What’s in a name?* U.S. Department of Health and Human Services; April, (2021).

[14] Dossett ML Fricchione GL Benson H. A new era for mind–body medicine. N Engl J Med. (2020) 382:1390–1. doi: 10.1056/NEJMp1917461, PMID: 32268025 PMC7486127

[15] Collins SV Hines AL. Stress reduction to decrease hypertension for black women: a scoping review of trials and interventions. J Racial Ethn Health Disparities. (2021) 9:2208–17. doi: 10.1007/s40615-021-01160-y, PMID: 34606073

[16] Johnson SB Goodnight BL Zhang H Daboin I Patterson B Kaslow NJ. Compassion-based meditation in African Americans: self-criticism mediates changes in depression. Suicide Life Threat Behav. (2018) 48:160–8. doi: 10.1111/sltb.12347, PMID: 28326598

[17] Peters MD Godfrey CM Khalil H McInerney P Parker D Soares CB. Guidance for conducting systematic scoping reviews. Int J Evid Based Healthc. (2015) 13:141–6. doi: 10.1097/XEB.0000000000000050, PMID: 26134548

[18] Haddaway NR Page MJ Pritchard CC McGuinness LA. PRISMA2020: an R package and shiny app for producing PRISMA 2020-compliant flow diagrams, with interactivity for optimised digital transparency and open synthesis. Campbell Syst Rev. (2022) 18:e1230. doi: 10.1002/cl2.1230, PMID: 36911350 PMC8958186

[19] Schünemann HJ Brennan S Akl EA Hultcrantz M Alonso-Coello P Xia J . The development methods of official GRADE articles and requirements for claiming the use of GRADE - a statement by the GRADE guidance group. J Clin Epidemiol. (2023) 159:79–84. doi: 10.1016/j.jclinepi.2023.05.010, PMID: 37211327

[20] Chang P-S Lu Y Nguyen CM Suh Y Luciani M Ofner S . Effects of qigong exercise on physical and psychological health among African Americans. West J Nurs Res. (2021) 43:551–62. doi: 10.1177/0193945920959067, PMID: 32942949 PMC8159432

[21] Johnson CC Taylor AG Anderson JG Jones RA Whaley DE. Feasibility and acceptability of an internet-based, African dance-modified yoga program for African-American women with or at risk for metabolic syndrome. J Yoga Phys Ther. (2014) 1:1000174. doi: 10.4172/2157-7595.1000174 PMC4292896 25593785

[22] Mama SK Bhuiyan N Chaoul A Cohen L Fagundes CP Hoover DS . Feasibility and acceptability of a faith-based mind-body intervention among African American adults. Transl Behav Med. (2020) 10:928–37. doi: 10.1093/tbm/iby114, PMID: 30476343 PMC7753004

[23] Zhou YE Jackson CD Oates VJ Davis GW Davis C Takizala ZM . Refining a church-based lifestyle intervention targeting African-American adults at risk for Cardiometabolic diseases: a pilot study. Open J Epidemiol. (2017) 7:96–114. doi: 10.4236/ojepi.2017.72009, PMID: 33457107 PMC7808719

[24] Schneider RH Grim CE Rainforth MV Kotchen T Nidich SI Gaylord-King C . Stress reduction in the secondary prevention of cardiovascular disease: randomized, controlled trial of transcendental meditation and health education in blacks. Circulation. (2012) 5:750–8. doi: 10.1161/CIRCOUTCOMES.112.967406, PMID: 23149426 PMC7269100

[25] Schneider RH Myers HF Marwaha K Rainforth MA Salerno JW Nidich SI . Stress reduction in the prevention of left ventricular hypertrophy: a randomized controlled trial of transcendental meditation and health education in hypertensive African Americans. Ethn Dis. (2019) 29:577–86. doi: 10.18865/ed.29.4.577, PMID: 31641325 PMC6802172

[26] Cox TL Krukowski R Love SJ Eddings K DiCarlo M Chang JY . Stress management–augmented behavioral weight loss intervention for African American women: a pilot, randomized controlled trial. Health Educ Behav. (2012) 40:78–87. doi: 10.1177/1090198112439411, PMID: 22505570

[27] Bernstein AM Gendy G Rudd N Doyle J Fay S Moffett K . Management of prediabetes through lifestyle modification in overweight and obese African-American women: the fitness, relaxation, and eating to stay healthy (FRESH) randomized controlled trial. Public Health. (2014) 128:674–7. doi: 10.1016/j.puhe.2014.04.005, PMID: 24996961

[28] Woods-Giscombe CL Gaylord SA Li Y Brintz CE Bangdiwala SI Buse JB . A mixed-methods, randomized clinical trial to examine feasibility of a mindfulness-based stress management and diabetes risk reduction intervention for African Americans with prediabetes. Evid Based Complement Altern Med. (2019) 2019:1–16. doi: 10.1155/2019/3962623, PMID: 31511777 PMC6710811

[29] Waldron EM Burnett-Zeigler I. The impact of participation in a mindfulness-based intervention on posttraumatic stress symptomatology among black women: a pilot study. Psychol Trauma. (2022) 14:29–37. doi: 10.1037/tra0001107, PMID: 34435817

[30] Burnett-Zeigler I Hong S Waldron EM Maletich C Yang A Moskowitz J. A mindfulness-based intervention for low-income African American women with depressive symptoms delivered by an experienced instructor versus a novice instructor. J Altern Complement Med. (2019) 25:699–708. doi: 10.1089/acm.2018.0393, PMID: 30912681

[31] Hong S Satyshur MD Burnett-Zeigler I. The association of mindfulness and depression stigma among African American women participants in a mindfulness-based intervention: a pilot study. Transcult Psychiatry. (2022) 60:244–54. doi: 10.1177/13634615221076709, PMID: 35505619

[32] Okhomina VI Seals SR Anugu P Adu-Boateng G Sims M Marshall GD Jr. Adherence and retention of African Americans in a randomized controlled trial with a yoga-based intervention: the effects of health promoting programs on cardiovascular disease risk study. Ethn Health. (2020) 25:812–24. doi: 10.1080/13557858.2018.1458073, PMID: 29609480

[33] Smith B Metzker K Waite R Gerrity P. Short-form mindfulness-based stress reduction reduces anxiety and improves health-related quality of life in an Inner-City population. Holist Nurs Pract. (2015) 29:70–7. doi: 10.1097/HNP.0000000000000075 25658929

[34] Scott-Sheldon LAJ Gathright EC Donahue ML Balletto B Feulner MM DeCosta J . Mindfulness-based interventions for adults with cardiovascular disease: a systematic review and Meta-analysis. Ann Behav Med. (2020) 54:67–73. doi: 10.1093/abm/kaz020, PMID: 31167026 PMC6922300

[35] Watt M Hyde A Spence JC Wright GM Vander Well S Johnson E . The feasibility and acceptability of an online mind-body wellness program for patients with primary biliary cholangitis. Can Liver J. (2023) 6:314–31. doi: 10.3138/canlivj-2022-0045, PMID: 38020194 PMC10652984

[36] Gravesande J Almeida de Oliveira L Malik N Vrkljan B Zheng R Gardner PM . Feasibility, usability, and acceptability of online mind-body exercise programs for older adults: a scoping review. J Integr Complement Med. (2023) 29:538–49. doi: 10.1089/jicm.2022.0822, PMID: 36944159

[37] Chu P Gotink RA Yeh GY Goldie SJ Hunink MG. The effectiveness of yoga in modifying risk factors for cardiovascular disease and metabolic syndrome: a systematic review and meta-analysis of randomized controlled trials. Eur J Prev Cardiol. (2016) 23:291–307. doi: 10.1177/2047487314562741, PMID: 25510863

[38] Lopez Abel B Martinez-Soto MI Couce ML. Integrative cardiology-state of the art of mind body therapies for the treatment of cardiovascular disease and risk factors. AIMS Med Sci. (2018) 5:80–9. doi: 10.3934/medsci.2018.1.80

[39] Leung LY Chan AW Sit JW Liu T Taylor-Piliae RE. Tai chi in Chinese adults with metabolic syndrome: a pilot randomized controlled trial. Complement Ther Med. (2019) 46:54–61. doi: 10.1016/j.ctim.2019.07.008, PMID: 31519288

[40] Dar T Radfar A Abohashem S Pitman RK Tawakol A Osborne MT. Psychosocial stress and cardiovascular disease. Curr Treat Options Cardiovasc Med. (2019) 21:23. doi: 10.1007/s11936-019-0724-5, PMID: 31028483 PMC6568256

[41] Vergeer I Bennie JA Charity MJ Harvey JT van Uffelen JGZ Biddle SJH . Participation trends in holistic movement practices: a 10-year comparison of yoga/Pilates and t'ai chi/qigong use among a national sample of 195,926 Australians. BMC Complement Altern Med. (2017) 17:296. doi: 10.1186/s12906-017-1800-6, PMID: 28587599 PMC5461749

[42] Davidson EM Liu JJ Bhopal R White M Johnson MR Netto G . Behavior change interventions to improve the health of racial and ethnic minority populations: a tool kit of adaptation approaches. Milbank Q. (2013) 91:811–51. doi: 10.1111/1468-0009.12034, PMID: 24320170 PMC3876191

[43] Biggers A Spears CA Sanders K Ong J Sharp LK Gerber BS. Promoting mindfulness in African American communities. Mindfulness (N Y). (2020) 11:2274–82. doi: 10.1007/s12671-020-01480-w, PMID: 33584869 PMC7880239

[44] National Institue on Minority Health and Health Disparities. *Scientific goals, research strategies, and priority areas: U.S. Department of Health and Human Services*. (2024). Available at: https://www.nimhd.nih.gov/about/strategic-plan/nih-strategic-plan-scientific-goals-research-strategies-priority-areas.html#goal3.

[45] Mehta LS Velarde GP Lewey J Sharma G Bond RM Navas-Acien A . Cardiovascular disease risk factors in women: the impact of race and ethnicity: a scientific statement from the American Heart Association. Circulation. (2023) 147:1471–87. doi: 10.1161/CIR.0000000000001139, PMID: 37035919 PMC11196122

[46] Office of Disease Prevention and Health Promotion. *Improve cardiovascular health in adults — HDS-01 — Health People 2030: U.S. Department of Health and Human Services*. (n.d.) Available at: https://odphp.health.gov/healthypeople/objectives-and-data/browse-objectives/heart-disease-and-stroke/improve-cardiovascular-health-adults-hds-01.

[47] Office of Disease Prevention and Health Promotion. *Reduce the proportion of adults with high blood pressure — HDS-04 — Health People 2030: U.S. Department of Health and Human Services*. (n.d.) Available at: https://odphp.health.gov/healthypeople/objectives-and-data/browse-objectives/heart-disease-and-stroke/reduce-proportion-adults-high-blood-pressure-hds-04.

[48] Office of Disease Prevention and Health Promotion. *Reduce the number of diabetes cases diagnosed yearly — D-01 — Health People 2030: U.S. Department of Health and Human Services*. (n.d.) Available at: https://odphp.health.gov/healthypeople/objectives-and-data/browse-objectives/diabetes/reduce-number-diabetes-cases-diagnosed-yearly-d-01.

[49] Office of Disease Prevention and Health Promotion. *Reduce the proportion of adults who do no physical activity in their free time — PA-01 — Healthy People 2030: U.S. Department of Health and Human Services*. (n.d.). Available at: https://odphp.health.gov/healthypeople/objectives-and-data/browse-objectives/physical-activity/reduce-proportion-adults-who-do-no-physical-activity-their-free-time-pa-01.

[50] National Institutes of Health. *NIH-Wide Strategic Plan for Diversity, Equity, Inclusion, and Accessibility (DEIA): U.S. Department of Health and Human Services*. (2023). Available at: https://www.nih.gov/about-nih/nih-wide-strategic-plan-diversity-equity-inclusion-accessibility-deia.

